# Vice Sets Its Seal

**Published:** 1892-12

**Authors:** 


### VICE SETS ITS SEAL.

A writer on social purity says that even impure thoughts, habitually indulged, may be revealed by a permanent change in the expression of the countenance. Though a man imagines that no one suspects what is passing in his mind, the lines of his face may become so altered by foul imaginations that pure women shrink when he speaks to them, and even men of the world say they "somehow don't like that fellow." We read that "the books shall be opened." They are being opened now, for the record of a man's inmost thoughts are legibly spread on the open page of his countenance, and though he thinks not of it, he may be already delivered over to "everlasting shame and contempt."

